# Editorial: Immune determinants of T cell responses to recall or *de novo* antigens

**DOI:** 10.3389/fimmu.2026.1995690

**Published:** 2026-09-29

**Authors:** María Marcela Barrio, Krystelle Nganou, Ashish Sharma, Angela Wahl

**Affiliations:** 1 Centro de Investigaciones Oncológicas, Fundación Cáncer FUCA, Buenos Aires, Argentina; 2 Department of Medicine 3, Uniklinikum Erlangen, Erlangen, Germany; 3 Pathology Advanced Translational Research Unit, Department of Pathology & Laboratory Medicine, Emory University School of Medicine, Atlanta, GA, United States; 4 Department of Microbiology, University of Alabama at Birmingham, Birmingham, AL, United States

**Keywords:** *de novo*, Gamma Delta (γδ) T cells, recall (memory), salmonella, SARS-CoV-2, T cell response, tumor neoantigen, vaccination

T cells play a central role in protective immunity against pathogens and are increasingly recognized as critical mediators of vaccine-induced immune responses. However, unlike antibody responses, T-cell immunity is less frequently assessed due to methodological complexity, limited standardization, and lower assay sensitivity, restricting the identification of T-cell-based correlates of protection. Most vaccines for infectious diseases are designed to elicit neutralizing antibody responses to prevent infection. However, T cells can clear infection when vaccine-induced antibodies fail to prevent it. Growing evidence indicates that baseline immune status influences the magnitude and durability of antigen-specific T-cell responses following infection or vaccination. The development of therapeutic cancer vaccines has further emphasized the need to characterize T-cell responses to tumor-associated antigens and neoantigens. Nevertheless, substantial inter-individual variability, influenced by aging and chronic viral infections, remains poorly understood. This Research Topic explores the immune determinants shaping T-cell responses during infection and vaccination.

In this Research Topic, which contains eight articles, five focus on SARS-CoV-2 infection.

Florian et al. present a longitudinal study of T cell immunity assessed in a cohort of SARS-CoV-2 vaccinated and infected individuals monitored over three years. The authors show that cross-reactive T cell immunity to seasonal coronaviruses strongly influences infection outcomes in COVID-19 patients, and that this spike-cross-reactive T cell immunity extends well beyond the initial SARS-CoV-2 exposure, contributing to a long-lived memory T cell pool that responds to highly mutated Omicron variants. In another article, Aritsu et al. showed that pyroglutamylation at the N-terminus of an immunodominant HLA-A*24:02-restricted epitope of the SARS-CoV-2 spike protein increases protease resistance and enhances T cell responses and protection in transgenic mouse studies. Their results suggest that subtle modifications of antigenic peptides can enhance T cell immunity in SARS-CoV-2 infection, with potential translation into the design of new T cell-based vaccines against viral infections. Ugel et al. further report on the immune dynamics of the anti-spike cellular T-cell response following SARS-CoV-2 infection and/or vaccination among healthcare workers. The study demonstrates that CD8-restricted immunity induced by vaccination follows a dynamic pattern, where the percentages of CD154+ and spike-specific CD45RO+ CD8 T cells may contribute to protection against infection. These findings support incorporating spike-specific T cell responses into larger studies to validate their contribution to protective immunity against SARS-CoV-2. Parsons et al, investigated T cell receptor usage in a well-characterized longitudinal COVID-19 cohort, identifying total TCR repertoire patterns associated with disease severity as well as CD8 TCR patterns associated with disease and vaccination status. Importantly, the authors uncover patterns of Spike-specific TCR expansion and contraction associated with vaccination, and non-Spike-specific epitope targets associated with disease severity. Wang et al. delved into the dynamic characteristics of SARS-CoV-2-specific B-cell and T-cell responses in people living with HIV (PLWH) vaccinated with the inactivated SARS-CoV-2 vaccine CoronaVac. This study revealed that the peak frequencies of Spike-specific B-cell and T-cell responses in PLWH are lower than those in HIV-negative controls. The authors emphasize that the weak SARS-CoV-2-specific B-cell and T-cell responses induced in PLWH have implications for clinical decision-making and public health policy regarding SARS-CoV-2 infection. Altogether, these five studies on SARS-CoV-2 clarify our understanding of protective mechanisms and disease outcome predictors, providing knowledge relevant in light of ongoing viral evolution.

In a complementary vaccine perspective, van der Most and Leroux-Roels discuss the role of CD4 T-cell responses in the protection conferred by many infectious disease vaccines, mainly those formulated with the AS01 adjuvant. While dissecting the vaccine-induced CD4 T-cell responses across several studies, the authors identify a pattern in which the capacity of antigen-specific vaccine-induced CD4 T cells to produce interferon-γ appears to depend on increased levels of prior exposure, as well as pre-existing memory T cells. This contribution could inform research to improve vaccine development and may also support analyses of vaccine efficacy studies. Using a multi-omics approach to novel antigen identification, Carri et al. investigated T-cell immune responses elicited by adjuvant vaccination with the cellular vaccine VACCIMEL, in human melanoma patients. The authors show that after receiving VACCIMEL, patients’ immune systems mount concurrent responses to a diverse array of antigens, including shared antigens such as tumor-associated antigens, private tumor-derived neoantigens, and public neoantigens present in the vaccine, as well as VACCIMEL-exclusive antigens derived from polymorphisms and somatic mutations. Furthermore, an extensive analysis of epitope features revealed that T-cell responses against VACCIMEL correlate with antigen expression in the vaccine cells.

Lastly, Tetzlaff et al. present research on avian immune responses, using a novel γδ T cell-deficient chicken line to address the immunological role of this T cell population in response to *Salmonella Enteritidis* infection. The authors show that γδ T cells play a pivotal role in mediating early pathogen control in chickens, and that in their absence, expansion of other non-γδ T cell subsets and functional adaptations of the immune system appear as compensatory mechanisms, shedding light on immune plasticity.

The contributions included in this Research Topic highlight the increased recognition of T cells as central actors in immune responses against infections and in cancer immunotherapy. More research in this field is warranted to leverage the immune system for the rational design of better immunotherapeutic tools.

